# Sulfur Modifications of the Wobble U_34_ in tRNAs and their Intracellular Localization in Eukaryotic Cells

**DOI:** 10.3390/biom7010017

**Published:** 2017-02-18

**Authors:** Yumi Nakai, Masato Nakai, Takato Yano

**Affiliations:** 1 Departments of Biochemistry, Osaka Medical College, 2-7 Daigaku-cho, Takatsuki Osaka 569-8686, Japan; med013@osaka-med.ac.jp; 2 Institute for Protein Research, Osaka University, 3-2 Yamada-oka, Suita Osaka 565-0871, Japan; nakai@protein.osaka-u.ac.jp

**Keywords:** the wobble uridine (U_34_), thio-modification of U_34_ (s^2^U_34_), iron-sulfur (Fe/S), cluster, cytosolic tRNA (cy-tRNA), mitochondrial tRNA (mt-tRNA)

## Abstract

The wobble uridine (U_34_) of transfer RNAs (tRNAs) for two-box codon recognition, i.e., tRNA^Lys^_UUU_, tRNA^Glu^_UUC_, and tRNA^Gln^_UUG_, harbor a sulfur- (thio-) and a methyl-derivative structure at the second and fifth positions of U_34_, respectively. Both modifications are necessary to construct the proper anticodon loop structure and to enable them to exert their functions in translation. Thio-modification of U_34_ (s^2^U_34_) is found in both cytosolic tRNAs (cy-tRNAs) and mitochondrial tRNAs (mt-tRNAs). Although l-cysteine desulfurase is required in both cases, subsequent sulfur transfer pathways to cy-tRNAs and mt-tRNAs are different due to their distinct intracellular locations. The s^2^U_34_ formation in cy-tRNAs involves a sulfur delivery system required for the biosynthesis of iron-sulfur (Fe/S) clusters and certain resultant Fe/S proteins. This review addresses presumed sulfur delivery pathways for the s^2^U_34_ formation in distinct intracellular locations, especially that for cy-tRNAs in comparison with that for mt-tRNAs.

## 1. Introduction

To date, over 100 different modifications have been found in RNA molecules from all domains of life [1] and, in particular, a variety of post-transcriptional modifications found in transfer RNAs (tRNAs) are known to have crucial roles for maintaining their structural stability and decoding accuracy, for effective codon recognition, and to completely contribute to protein translation [2,3,4].

Thio-modification in tRNAs involves the incorporation of sulfur into the carbon ring of purine or pyrimidine that occurs as a direct exchange of oxygen to sulfur [1]. Thio-modification of wobble uridine (U_34_) in tRNA is found at the second (C2) and/or fourth positions (C4) of the pyrimidine ring (termed s^2^U and s^4^U, respectively), and the s^2^U is often found in all domains of life, whereas the s^4^U is only found in eubacteria [5]. The mechanism behind the delivery of a sulfur atom to the U_34_ poses an intriguing problem because such intracellular sulfur delivery and transfer systems remain incompletely understood.

The s^2^ modification (thio-modification) is found at the U_34_ of the two-split box codon (lysine, glutamate, and glutamine) tRNAs. As mentioned above, thio-modification of U_34_ is found in all domains of life, and particularly in eukaryotes, this modification is found not only in cytosolic tRNAs (cy-tRNAs), but also in mitochondrial genome-encoded mitochondrial tRNAs (mt-tRNAs). The final products of the biosynthesized s^2^U_34_ have identical structures in tRNAs in both locations; however, the s^2^U_34_ formation pathways and participating components appear to be different. In particular, concerning the thio-modification of cy-tRNAs, to date, many proteins have been explored for their involvement in the sulfur delivery to the s^2^U_34_ of cy-tRNAs in yeast (*Saccharomyces cerevisiae*), nematode (*Caenorhabditis elegans*), and human cells [2]. Nevertheless, overall understanding of this process remains incomplete. Thus, in the present review, we point out remaining questions regarding thio-modification in different subcellular locations in eukaryotic cells, particularly emphasizing the intracellular sulfur transfer pathway to cy-tRNAs in eukaryotes that should be revealed in the future.

An additional topic in relation to the thio-modification of cy-tRNAs included in the present review is “hyper-modification” of U_34_. The U_34_ in both cy- and mt- tRNAs possess a methyl-derivative modification at C5 in addition to thio-modification at C2 of the same uridine [6]. This “hyper-modification” of U_34_ would be an additional important factor to maintain thio-modification of tRNAs. Thus, the relation between these modifications at U_34_ is also discussed.

## 2. Cysteine Desulfurase Nfs1 and Thio-Modification of tRNA

**Figure 1:**
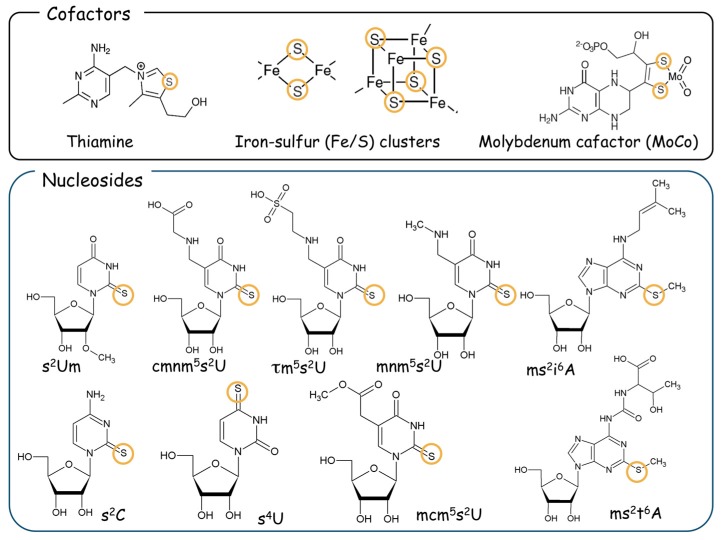
Sulfur-containing small molecules. Many biocofactors such as thiamine, iron-sulfur (Fe/S) clusters, and molybdenum cofactor (MoCo) contain sulfur atoms in their structure (upper column). Various sulfur-containing nucleosides are also identified. For example, the 2-thiocytidine derivatives are 2-thiocytidine (s^2^C) found in bacteria and 2-thio-2′-*O*-methyluridine (s^2^Um) found in humans. 4-thiouridine (s^4^U) is found in bacteria. Various types of the hyper-modified uridine, such as 5-carboxymethylaminomethyl-2-thiouridine (cmnm^5^s^2^U), 5-methylaminomethyl-2-thiouridine (mnm^5^s^2^U), 5-taurinomethyl-2-thiouridine (τm^5^s^2^U), and 5-methoxycarbonylmethyl-2-thiouridine (mcm^5^s^2^U) are found at U_34_. Various methylthio-adenosine derivatives, such as 2-methylthio- *N*6-isopentenyladenosine (ms^2^i^6^A) and 2-methylthio-*N*6-threonylcarbamoyladenosine (ms^2^t^6^A), are also found in both bacteria and eukaryotes. Sulfur atoms incorporated into the molecule in the biosynthetic pathway are shown with circles.

**Figure 2:**
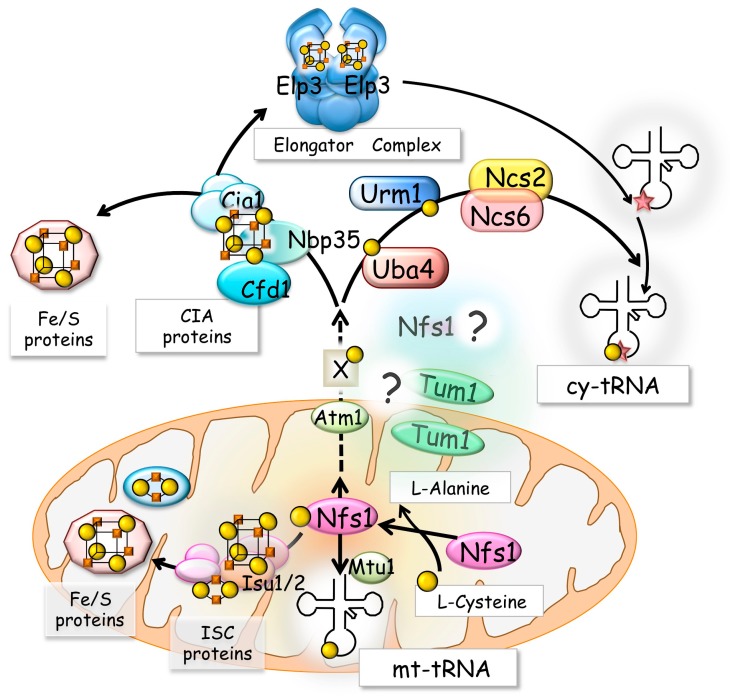
Involvement of the l-cysteine desulfurase Nfs1 in transfer RNA (tRNA) modification and iron-sulfur (Fe/S) cluster biosynthesis in *Saccharomyces cerevisiae*. The l-cysteine desulfurase Nfs1, identified as a member of the iron-sulfur cluster biogenesis (so-called “ISC”) proteins, is essential for the biogenesis of s^2^U_34_ in both cytosolic transfer RNAs (cy-tRNAs) and mitochondrial transfer RNAs (mt-tRNAs). Mtu1 is a mitochondrial tRNA-specific 2-thiouridylase. Comparing to the case of humans, existence of the cytosolic Nfs1 and the dual location of Tum1 are unclear in yeast (see text in detail). Cytosolic Fe/S protein maturation requires mitochondrial Nfs1 and the cytosolic iron-sulfur cluster assembly (so-called “CIA”) proteins, Cfd1, Nbp35, and Cia1. A putative unknown sulfur carrier to be exported from mitochondria via a membrane-bound protein Atm1 is shown as X. Elongator complex function as a dimer of the complex of Elongator complex 1-6 (ELP1-6) proteins, and function in the *C5* modification (marked with a star) of U_34_. Both a cytosolic ubiquitin-like protein (UBL), ubiquitin-related modifier 1 (Urm1), and its partner sulfurtransferase, ubiquitin-activating enzyme-like protein 4 (Uba4), are required for the formation of s^2^U_34_ in cy-tRNAs. Ncs6 and Ncs2 are also involved in the s^2^U_34_ formation in cy-tRNAs. The sulfur atoms delivered to the iron-sulfur (Fe/S) clusters and s^2^U_34_ in both mitochondrial (mt-) and cytosolic (cy-) tRNAs are shown with filled circles. Fe atoms of Fe/S clusters are shown with filled squares. See text in detail.

Bacterial l-cysteine desulfurase IscS is known to participate in the formation of s^2^U_34_ [7,8]. l-cysteine desulfurase (EC 2.8.1.7), is a pyridoxal-5′-phosphate (PLP)-containing protein. The substrate l-cysteine is initially bound to the PLP to form an intermediate with a Schiff base, and then a highly-conserved active site cysteine residue of this enzyme attacks the γ-sulfhydryl group of the PLP-cysteine intermediate, resulting in the abstraction of γ-SH from the intermediate to form a persulfide on the active-site cysteine residue of the enzyme. Such a persulfide formation in this enzyme is indispensable for biogenesis of the sulfur-bound small biomolecules (Figure 1).

l-Alanine is formed from the substrate l-cysteine, and a sulfur atom removed from the substrate is attached to the active site of the enzyme [5,8]. The physiological functions of this enzyme are to form a protein-bound persulfide as an intermediate and to provide the persulfide-derived sulfur (sulfan sulfur) to form various intracellular small sulfur-containing molecules such as iron-sulfur (Fe/S) clusters, molybdopterin, thiamine thiazole, and nucleotides [9,10]. Likewise for bacteria, eukaryotic l-cysteine desulfurase Nfs1 is essential for the biogenesis of s^2^U_34_ in both cy-tRNAs and mt-tRNAs [11,12,13,14] (Figure 2). Eukaryotic Nfs1 is also identified as a member of the *iron-sulfur cluster biogenesis* (so-called “ISC”) proteins [15] and functions as a sulfur donor for the cluster scaffold in mitochondria [16].

Yeast Nfs1 is essential for growth and is mainly located in the mitochondrial matrix [17,18]. When Nfs1 is depleted, thio-modifications of both mt-tRNAs and cy-tRNAs are strongly reduced, although thio-modified mt-tRNAs decrease rather faster than those of cy-tRNAs in yeast cells [11]. The fact that Nfs1 contributes to the formation of s^2^U_34_ in both intracellular locations appears consistent with the observation that yeast mitochondrial Nfs1 is also required for initial provision of sulfan sulfur in both mitochondrial and cytosolic Fe/S cluster biogenesis [19].

The mitochondrial Fe/S cluster formation system has been well studied and many proteins have been shown to be involved in this system [15] (Figure 2). Sulfur provided by the Nfs1 function is transferred to the scaffold IscU1/IscU2 hetero complex to form Fe/S clusters, following which it is delivered to various apo-Fe/S proteins after several steps of cluster transfer. On the other hand, a detailed molecular mechanism of the formation of s^2^U_34_ in mt-tRNAs remains to be elucidated. The s^2^U_34_ in mt-tRNAs is hyper-modified to cmnm^5^s^2^U (5-carboxymethylaminomethyl 2-thiouridine) and τm^5^s^2^U (5-taurinomethyl2-thiouridine) in yeast and humans, respectively [20,21,22]. Mtu1, a mitochondrial tRNA-specific 2-thiouridylase, is identified to be responsible for the generation of cmnm^5^s^2^U_34_ tRNA in yeast and τm^5^s^2^U_34_ in mammals [14] (Figure 2). Mtu1 is a eukaryotic orthologue of bacterial MnmA [14]. *Escherichia coli* MnmA is involved in the formation of s^2^U_34_ in the *E. coli* sulfur-relay system for tRNA thio-modification together with the TusA, TusBCD, and TusE proteins and MnmE [23,24]. However, in the yeast or human cases, no bacterial (Tus) orthologues have been identified to date, and mitochondrial ISC proteins except for Nfs1 appear not to be involved in the thio-modification of mt-tRNAs [11]. Therefore, an as-yet-unidentified sulfur transfer system that links mitochondrial Nfs1 and Mtu1 functions might exist in mitochondria.

The formation of s^2^U_34_ in yeast cy-tRNAs is also strongly reduced when the expression of Nfs1 is completely repressed [11]. The explanation for the contribution of yeast Nfs1 to the formation of s^2^U_34_ in cytosol remains a focus of discussion, since, although Nfs1 is mainly localized in the mitochondrial matrix as mentioned above, the existence of extra-mitochondrial Nfs1 has been proposed [25,26]. In the case of the yeast *S. cerevisiae*, circumstantial evidence for possible dual localization of Nfs1 in the nuclear compartment in addition to the mitochondrial matrix was previously demonstrated by a genetic complement study [25] and by a mitochondrial peptidase Icp55-using experiment [26]. However, any role of yeast Nfs1 in the nucleus remains unclear. Yeast Nfs1 is a single copy nuclear gene product carrying a mitochondrial targeting signal that is cleaved off in the mitochondrial matrix. If any functional Nfs1 exists in cytosol, it might facilitate the thio-modification of cy-tRNAs, possibly for more direct initial provision of sulfan sulfur in cytosol. However, Nfs1 has not yet been biochemically identified in cytosol in yeast, nor has any sulfur transfer system using putative cytosolic Nfs1 been identified in vivo. Alternatively, if yeast Nfs1 is exclusively localized in mitochondria, but is required for the delivery of sulfur to cy-tRNAs, such hypothetical existence of functional Nfs1 in cytosol is not necessary; however, a certain sulfan sulfur delivery system from the mitochondrial matrix to cytosol must absolutely be required.

Differences might exist in human cases. Small amounts of human Nfs1 (NFS1) and other mitochondrial ISC proteins, ISCU (human orthologue of yeast IscU1/U2), NFU1, heat shock protein 20 (HSC20), and FXN (human orthologue of yeast frataxin), are detected in human cytosol [18,27,28,29,30,31,32,33]. When the plasmid-borne NFS1 gene was introduced and expressed in HeLa cells, NFS1 protein was detected in the cytosolic space by microscopic observation [34] and biochemical analyses [18]. However, it should be noted that cytosolically-expressed human NFS1 appears not to support the cytosolic Fe/S cluster assembly de novo without functional mitochondrially-localized NFS1 [16]. Therefore, it remains critically important to determine whether only cytoplasmic NFS1, rather than mitochondrial NFS1, is required for the formation of s^2^U_34_ in the human cytosol. Considering all of the above, Nfs1/NFS1 functions in both mitochondrial and cytosolic tRNAs thio-modification, most likely by providing the sulfur of the s^2^U_34_; however, the exact sulfur delivery pathway to tRNAs in distinct subcellular locations is complex and remains to be elucidated.

## 3. The Cytosolic UBL-UBA System and Thio-Modification of tRNA

**Figure 3:**
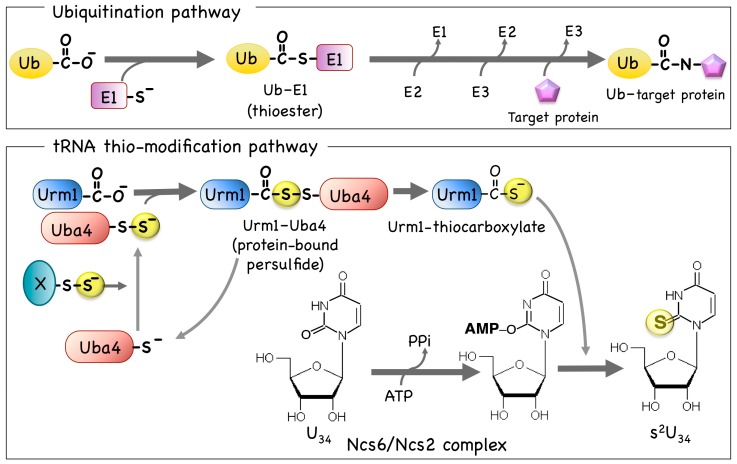
The UBL—ubiquitin-activating enzyme (E1)-like protein system related to the thio-modification of cytosolic transfer RNA (tRNA). The protein modifier system of ubiquitination found in eukaryotes (upper column) is compared with a plausible model for the sulfur transfer between Uba4 and Urm1, which is for the s^2^U_34_ formation in yeast cytosolic tRNA (lower column). In the ubiquitination pathway, ubiquitin (Ub) is first bound to the UBA protein E1 (a sulfhydryl of an active cysteine residue is shown in a yellow circle) and then, after several reactions, is finally bound to form a thioester bond. In the thio-modification pathway, a UBL protein Urm1 is first bound to the partner UBA protein, Uba4. A complex of Ncs6 and Ncs2 (the Ncs6/Ncs2 complex) is thought to be involved in the last step of the tRNA recognition and thio-modification of cy-tRNAs. Sulfur atoms transferred in this system are shown with filled circles.

In the ubiquitin (Ub)-dependent protein degradation system, Ub is attached to its final target protein after several steps of the activated-Ub transfer between Ub and its partner UBA (ubiquitin activating enzyme-like proteins), that is, E1 (the ubiquitin activating enzyme), E2, and E3 enzymes. In Ub activation with its partner UBAs, the carboxyl terminal glycine of Ub binds to the active site cysteine residue of UBA by forming a thioester bond [35] (Figure 3). Many different ubiquitin-like protein (UBL)-UBA pairs are known to be involved in a variety of intracellular signal transduction systems [35] other than the protein degradation. Such UBLs show similar β-grasp fold structures and possess conserved glycine-glycine residues at the carboxyl terminal of the proteins [35]. UBA binds its partner UBL by a thioester bond, following which the UBL is transferred to another UBA protein of the same signal transduction stream, after which it is finally attached to the target protein [35]. The first reaction of the UBL-UBA system is thioester bond formation between the C-terminal glycine of UBL and the cysteine residue of the partner UBA [35]. Yeast ubiquitin related modifier 1 (Urm1) and its partner Uba4 form a unique UBL-UBA protein pair that was revealed to be required for the formation of s2U34 in cy-tRNAs [36,37,38,39,40]. A unique characteristic of Urm1 is that its final structure in sulfur transfer to the formation of s2U34 is not a protein-bound form, but rather its terminal thiocarboxylate formation, whereas the final form of other UBLs are protein-bound forms [35]. Interestingly, similar sets of the proteins are found in bacterial sulfur transfer systems such as thiazole formation in thiamine biosynthesis and molybdopterin (MPT) formation in molybdenum cofactor (MoCo) biosynthesis [36]. Thus, the Urm1-Uba4 system is thought to be an ancient sulfur transfer system that originally functioned in the bacterial sulfur transfer system found in the biogenesis of thiamine thiazole or MPT [36,39,41] (Figure 3).

**Figure 4:**
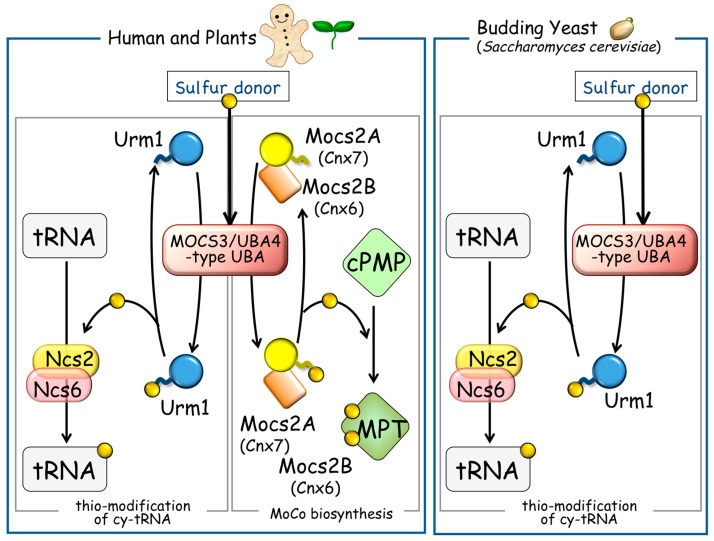
Sulfur delivery to the U_34_ in cy-tRNAs and for molybdopterin (MPT) biosynthesis in eukaryotes. Multicellular eukaryotes (human and plant) possess two types of ubiquitin-like (UBL proteins, for MoCo biosynthesis, and for the thio-modification of tRNA, both of which associate with the common Uba4/MOCS3-type UBA proteins (left panel). On the other hand, the yeast *Saccharomyces cerevisiae* contains no MoCo biosynthesis-related proteins and only Urm1 for thio-modification of tRNAs associates to Uba4 (right panel). Sulfur atoms to be delivered are shown with filled circles.

The Uba4/MOCS3-type UBA proteins including yeast Uba4 are found in all domains of life [42,43]. Human and plant Uba4/MOCS3-type UBA proteins, namely molybdenum cofactor synthesis protein 3 (MOCS3) and Cnx5, respectively, were firstly identified as sulfurtransferases required to construct an MPT in MoCo biosynthesis [44]. MoCo biosynthesis is also found in various organisms, but not in some fungi including *S. cerevisiae* and many parasitic protozoa [44,45]. Therefore, Uba4 appears to be a sulfurtransferase specific to the thio-modification of cy-tRNAs in the yeast *S. cerevisiae*. On the other hand, the Urm1-type UBL proteins are also ubiquitously found in eukaryotes and archaea [42,43]; however, an additional UBL protein (molybdenum cofactor synthesis protein 2A (MOCS2A)-type UBL proteins) exists that is only found in MoCo-producing organisms. Plant Cnx7 belongs to the UBL of this type and is required for MoCo synthesis, but not for thio-modification of cy-tRNAs. Thio-modification in yeast *∆URM1* cells is restored only by the Urm1-type UBL protein Urm1 (*Arabidopsis thaliana* Urm11 and Urm12), but not by MOCS2A-type UBL protein Cnx7 [42]. In addition, human MOCS3 has the ability to transfer sulfur that is attached to its C-terminal rhodanese-like domain to two distinct acceptor proteins, MOCS2A and URM1 in human cells [13,37,38]. These results indicate the distinctive role of the Urm1-type and the MOCS-type UBL proteins [42] (Figure 4).

Yeast Uba4 and human MOCS3 contain the N-terminal MoeB/E1-like domain (MoeB is an *E. coli* orthologue of the human MOCS3) and the C-terminal rhodanese-like domain [36,46]. The N-terminal MoeB/E1-like domain of the Uba4/MOCS3-type UBA proteins can bind the C-terminally adenylated Urm1 via a thioester bond that is commonly found in the activation mechanism of various UBL proteins [35]. The C-terminal rhodanese-like domain of these Uba4/MOCS3-type UBA proteins can provide sulfur to Urm1 to ultimately form a thiocarboxylate at the conserved C-terminal glycine of Urm1 [46,47]. Although the sulfur transfer mechanism from Uba4 to Urm1 remains incompletely understood, by analogy, similar to the bacterial acyldisulfide linkage formation between ThiS and ThiF (tRNA sulfurtransferase F) [48,49,50] found in the biosynthesis of thiamine thiazole [51,52], a protein-bound persulfide might be formed as an intermediate structure in the sulfur transfer between Uba4 and Urm1.

A combination study of the split-enhanced green fluorescent protein system and fluorescence resonance energy transfer analysis showed that NFS1 without a mitochondrial localization signal can interact with MOCS3 in the cytosol of HeLa cells when the N-terminal signal-truncated NFS1 is ectopically expressed [34]. In addition, an in vitro biochemical assay showed that human NFS1 could transfer sulfur to the C-terminal domain of MOCS3 [34]. On the other hand, the Uba4 without its N-terminal MoeB/E1 domain cannot complement thio-modification of cy-tRNA, thereby indicating that the whole region of Uba4 is indispensable for the s^2^U_34_ formation in cy-tRNA in yeast [36]. Thus, the direct interaction between NFS1 with MOCS3 in the sulfur transfer in human cytosol in vivo remains to be investigated.

Yeast Tum1 (Yor251c) is a double rhodanese domain-containing sulfurtransferase. The rhodanese superfamily is a versatile sulfur carrier protein catalyzing sulfur transfer reactions in various metabolic and regulatory pathways [53,54]. The s^2^U_34_ formation in cy-tRNAs is partially reduced when the Tum1 gene is disrupted, indicating that a Tum1-mediated sulfur transfer to the s^2^U_34_ in cy-tRNAs is an alternative or redundant pathway besides another sulfur transfer pathway of thio-modification in cy-tRNAs [38,40,55]. Results of an in vitro sulfur transfer assay between Tum1 and Nfs1 [40] imply a possibility that extra-mitochondrial Nfs1 might exist and function in the sulfur transfer system of the cy-tRNA thio-modification pathway. However, the exact subcellular localization of yeast Tum1 remains unclear, and as described above, conclusive evidence for the existence of cytosolic Nfs1 and its direct involvement in cytosolic sulfur transfer reactions remains lacking in yeast cells [56,57]. Therefore, the Tum1-mediated sulfur transfer pathway in cytosol remains unclear. On the other hand, two forms of human Tum1 (TUM1) are found in both cytosol and mitochondria in HeLa cells [58], indicating that they may correspond to the observed dual localization of human Nfs1 (NFS1) [34]. Moreover, the isolated NFS1 and the rhodanese domain of Uba4/MOCS3 and of TUM1 can be interactive in vitro [59]. However, no direct evidence of mitochondrial-cytosolic sulfur transfer mediated by Tum1, nor the interaction between NFS1 and TUM1 in mitochondria, has yet been demonstrated. Thus, the direct involvement of TUM1 in s^2^U_34_ formation in human cy-tRNAs remains to be clarified.

## 4. Fe/S Proteins and the Formation of s^2^U_34_ in Cytosol

Cytosolic Fe/S protein maturation requires mitochondrial Nfs1 and the *cytosolic iron-sulfur cluster assembly* (so-called “CIA”) proteins [56,57,60]. Interestingly, thio-modification of cy-tRNAs in yeast requires the CIA proteins Cfd1, Nbp35, and Cia1 in addition to Nfs1 [12]. Considering that mitochondrial Nfs1 and CIA proteins are required for both the cytosolic Fe/S protein maturation and the cy-tRNA thio-modification, certain cytosolic Fe/S protein(s) might be directly involved in the formation of s^2^U_34_ in cy-tRNAs (Figure 2).

In vitro reconstitution of the mcm^5^s^2^U_34_ in tRNA shows that mcm^5^ formation at C5 is required for s^2^ formation [40]. Elongator complex (ELP), which is composed of six subunits (Elp1, Elp2, Elp3, Elp4, Elp5, and Elp6) is required for the formation of 5-methoxycarbonylmethyl (mcm^5^) or 5-carbamoylmethyl (ncm^5^) at C5 of U_34_ [61], and, intriguingly, the active subunit Elp3 contains a [4Fe-4S] cluster in addition to *S*-adenosylmethyonine [62]. Recently resolved structures of Elp3 and Elp4, Elp5, and Elp6 multi-subunit complex (Elp456) indicate the structural importance of Elp3 in tRNA binding ability and interaction with the (Elp456) [63,64]. Yeast *∆ELP3* cells exhibit a reduced thio-modification level of cytosolic tRNAs and lack the entire C5 modification of these tRNAs [36,37,40], resulting in reduced decoding efficiency in protein translation [61]. Therefore, Elp3 containing its Fe/S cluster might be a key enzyme to construct the entire “hyper-modified” U_34_ structure, including s^2^U_34_ formation of cytosolic tRNAs to fulfill their activity. Further studies to clarify the direct mechanistic relation of the Fe/S protein involving C5 modification and thio-modification of cytosolic tRNAs should be performed.

The recent finding from bacterial homologue of the yeast cytosolic 2-thiouridine synthetase (Ncs6) might show the implication of the requirement of another Fe/S protein in the s^2^U_34_ formation in cy-tRNAs. ThiI (bacterial s^4^U synthetase)-like protein recently found in *Methanococcus maripaludis* was designated as the putative bacterial Ncs6 by sequence similarity to eukaryotic Ncs6, and was revealed to contain a [3Fe-4S] cluster essential for the tRNA thio-modification of *M. maripaludis* [65,66]. Other yeast cytosolic 2-thiouridine synthetases Ncs2 and Ncs6, in addition to Uba4, Urm1, and Tum1, are necessary to construct the s^2^U_34_ in vitro [40]. Yeast Ncs6-lacking cells show depletion of thio-modification in cy-tRNAs [36,38,39,40]. Ncs6 contains a nucleotide binding motif (PP-loop motif) (SGGxDS) [55], resembling a catalytic motif of N-type ATP pyrophosphatases also found in bacterial MnmA (required for bacterial s^2^U_34_ formation [8,23]) and in bacterial ThiI (required for bacterial s^4^U_8_ formation [5,67]). In general, ATP pyrophosphatases are thought to be important to activate the target positions of pyrimidine bases by forming acyl-adenylate intermediates. In *Schizosaccharomyces pombe* and *C. elegans*, *C. elegans* Ncs6 orthologue (Ctu1) and *C. elegans* Ncs2 orthologue (Ctu2) are physically associated with the s^2^U_34_ formation of tRNAs [68]. *S. cerevisiae* Ncs6 and human orthologue ATP binding protein 3 (ATPBD3) bind Urm1 and URM1, respectively [69]. From these observations, a complex of Ncs6 and Ncs2 (the Ncs6/Ncs2 complex) is thought to be involved in the last step of the tRNA recognition and thio-modification of cy-tRNAs. Although which protein is an actual final sulfur donor to the U_34_ of the tRNA molecule and how the Ncs6/Ncs2 complex recognizes both Urm1 and the target tRNA and catalyzes the thio-modification reaction remain to be elucidated, investigations of the putative Fe/S-containing Ncs6 might reveal important implications of understanding the molecular mechanisms of thio-modification of eukaryotic cy-tRNAs [69].

## 5. Perspectives

The entire modification of U_34_, including thio-modification, is essential for the accuracy of codon-anticodon pairing and for effective decoding in protein translation, thereby contributing to a fine-tuning in protein productivity in cells [70]. Deficiency of the wobble U_34_ modifications in cy-tRNAs causes ribosome stalling at the A-site during translation [71], indicating that the wobble U_34_ modification including s^2^U_34_ formation is responsible for maintaining the efficiency of protein translation in diverse cellular functions. Structural stability in and around the anticodon loop would be important to guarantee such translation efficiency [72]. For example, when s^2^ and/or mcm^5^ modification at U_34_ together with A_37_ or ψ (pseudouridine)_38/39_ modifications are lacking, the morphologies of yeast cells are altered and growth defects are exhibited [73]. This indicates that the cooperative interaction of these tRNA modifications is essential for cell homeostasis. Deficiency of the wobble U_34_ modification might also affect degradation/decay of the tRNA, as has been proposed for other types of tRNA modifications [74].

Translation quality control in regard to the U_34_ modification of tRNA appears to influence diverse physiological effects in eukaryotic cells. When either of Uba4, Urm1, or Ncs6 is lacking, yeast cells cannot exhibit invasive growth as a stress response, which normally occurs under glucose starvation conditions [75]. Elevated growth temperature of yeast cells was shown to affect the tRNA modification status and cause changes to their morphology [74]. Yeast *NCS2*, *UBA4* (=*NCS3*), *NCS6*, and *ELP2* (=*NCS10*) were originally designated as genes whose mutations exhibit the synthetic lethality of *∆CLA4* cells [76]. Cla4 functions as a Cdc42-activated signal-transducing kinase and is involved in various physiological functions, including yeast morphology formation, budding, and metabolic control [77,78,79,80]. From this evidence, U_34_ modification in cytosolic tRNAs appears to be related to such variations in the morphological and physiological status of the cells.

In humans, pathogenic defects related to U_34_ modification in both mt-tRNAs and cy-tRNAs are reported. In regard to mitochondrial diseases that are known to involve mitochondrial tRNA modification [81], for example, patients with myoclonus epilepsy associated with ragged-red fibers (MERFF) syndrome bear a mutation (A8344G) in the T-arm of the mt-RNA gene, and the tRNA modification in the patients is impaired [82,83]. Another case is a reduction of respiration enzyme activity in the defect of a mitochondrial tRNA-modifying enzyme GTP-binding protein 3 (GTPBP3) that is involved in the C5 of U_34_ (τm^5^U) in mt-tRNAs [84]. In regard to pathogenic defects concerning the U_34_ modification of cy-tRNAs, in familial dysautonomia, for example, patients exhibit reduced levels of mcm^5^s^2^U in cy-tRNAs [85].

Many of the enzymes involved in tRNA thio-modification are also associated with additional physiological functions other than tRNA modification. Urm1 also functions as the “Urm1-related protein conjugation system” (so-called “urmylation”) besides thio-modification of tRNAs. urmylation is related to target of rapamycin (TOR) pathway-related signal transduction and cellular morphological changes under various nutritional stress conditions [74,86,87]. In addition, intracellular levels of sulfur-containing amino acids assimilated as nutrients can regulate the translation ability by changing the status of the thio-modification of tRNAs, thereby balancing the metabolic homeostasis in the cell [88]. Human URM1^-^decifient mutants result in the G1 arrest in cell cycle and exhibit multinuclear morphology [38]. The Uba4/MOCS3-type UBA proteins in multicellular eukaryotes have dual functions in sulfur transfer to tRNAs, as well as to MPT. Elongator complex, including Elp3, is also a multifunctional enzyme complex. Biosynthetic pathways of tRNA thio-modification and Fe/S protein biosynthesis in distinct intracellular locations appear to be deeply related and are indispensable for cell homeostasis.
